# Evaluating the effect of liver copper concentration on vaccine response in lightweight dairy-beef steers

**DOI:** 10.3168/jdsc.2025-0753

**Published:** 2025-05-16

**Authors:** Jacob A. Henderson, Olivia N. Genther-Schroeder, Jodi L. McGill, Stephanie L. Hansen

**Affiliations:** 1 Department of Animal Science, Iowa State University, Ames, IA 50011; 2 Land O' Lakes Inc., Gray Summit, MO 63039; 3 Department of Veterinary Microbiology and Preventative Medicine, Iowa State University, Ames, IA 50011

## Abstract

Dairy-beef crossbred calves often face greater copper exposure than native beef calves, which may affect their response to vaccines. To investigate this, 28 weaned dairy-beef steers (90.25 ± 1.28 kg; ∼8 wk old) were enrolled in a study to determine the effect of copper status on the antibody response to vaccine. Steers were blocked by BW into pens, and pens were randomly assigned to one of 2 dietary treatments: adequate liver Cu (ADE; fed no supplemental Cu) and excess liver Cu (HCU; supplemented with 20 mg Cu/kg DM). Steers were housed in pens of 6 to 8 animals for 82 d before d 0 of the vaccination period to create distinct liver Cu groupings. Seven days before initial vaccine dose, liver biopsies were collected, and steers were grouped into ADE (n = 13) and HCU (n = 15) treatments. Liver copper for ADE averaged 291 ± 24 mg/kg DM (range 240 to 376 mg/kg DM) and for HCU averaged 665 ± 23 mg Cu/kg DM (range 519 to 893 mg/kg DM). All calves were vaccinated on d 0 and boostered 21 d later with Bovilis Vista 5 SQ (Merck Animal Health, Madison, NJ) and an ovalbumin vaccine. Blood samples were collected on these days and again on d 49 to end the study. Serum antibody titers for bovine respiratory syncytial virus (BRSV), bovine herpesvirus 1 (BHV1), bovine viral diarrhea virus 1, and bovine viral diarrhea virus 2 (BVDV2) were analyzed by the Iowa State University Veterinary Diagnostic Laboratory (Ames, IA). Response to ovalbumin vaccination was determined using an ELISA. Plasma copper concentrations were determined on d 0, 21, and 49 via inductively coupled plasma-optical emissions spectrometry. Ovalbumin antibody production was determined by calculating the ratio of each sample to a positive control. Antibodies for BRSV, BHV1, and BVDV2 increased across days, but there were no differences by treatment, indicating that both treatments responded equally to the modified live vaccine. Ovalbumin antibodies were affected by treatment by day, where HCU had greater response to ovalbumin than ADE on d 21, but by d 49 ovalbumin antibodies were similar between treatments. These results suggest excessive liver copper concentrations may cause more rapid antibody production in response to certain antigens; however, more research is needed to determine the effects of increased liver Cu concentration on inflammation and immune system function.

## Highlights

• Adequate and high copper treatments responded equally to respiratory vaccination.
• Steers with high liver copper responded more quickly to ovalbumin vaccination.
• Inflammation may have led to increased antibody production in the high treatment.

## Graphical Abstract

**Figure:**
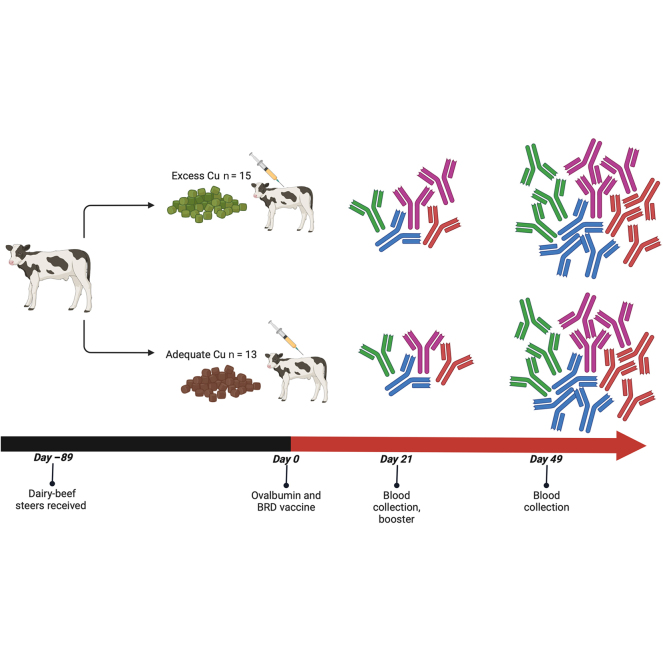
**Summary:** We studied the effect of excess liver copper concentration on plasma copper, serum haptoglobin, and antibody production in response to a respiratory (Bovilis Vista 5, Merck Animal Health, Madison, NJ) and an ovalbumin vaccine. Plasma copper and serum haptoglobin concentrations were not affected by treatment but decreased across days postvaccination. Titers for bovine respiratory syncytial virus, bovine herpesvirus 1, and bovine viral diarrhea virus 2 increased across days but were not affected by treatment. Steers with high liver copper had greater antibody production in response to ovalbumin 21 days postvaccination but were not different from steers with adequate liver copper by day 49 postvaccination. Figure created in BioRender.com.

Dairy-beef crossbred calves are exposed to much higher copper (Cu) concentrations than native beef calves. A study examining 39 dairy operations in California found the median Cu supplementation rate was 17.2 mg/kg diet DM, almost 8 mg/kg higher than NRC (2001) recommendations. Unsurprisingly, reports of Cu toxicosis in dairy cows have increased significantly in recent years (Kendall et al., 2015). Further, in a study examining necropsy reports of 601 calves up to 1 yr of age with causes of death unrelated to Cu toxicity, dairy calves had liver Cu concentrations 135 to 200 mg Cu/kg DM greater than beef calves, assuming the DM content of liver is 30% (Puschner et al., 2004). It is probable that excess Cu fed to dairy cows is translating to excess Cu in calves born to these cows, as Cu is transported to the fetus via the placenta (McArdle and Erlich, 1991). Despite this, calves born on commercial dairies are commonly fed milk replacer containing supplemental Cu, whereas nursing beef calves typically do not receive considerable Cu supplementation. Adequate Cu status based on liver parameters ranges from 125 to 600 mg Cu/kg DM in cattle (Kincaid, 1999); however, it has been noted that oxidative damage can occur in the liver at Cu concentrations as low as 400 mg Cu/kg DM (Strickland et al., 2019).

Although Cu plays an integral role in enzyme function and antioxidant capacity, excess Cu is known to contribute to oxidative stress. As the liver's capacity to safely store Cu is exceeded, free Cu^+^ ions are released, which contribute to the generation of reactive oxygen species (Bremner, 1998). This could result in impaired immune system function. Pocino et al. (1991) showed that feeding high concentrations of Cu to mice impaired cellular and humoral immune responses, and pro-inflammatory cytokine production in response to sheep red blood cells decreased as Cu intake increased. Elevated Cu status impairs the response to influenza vaccination in young men and reduces the percentage of circulating neutrophils and serum IL-2R (Turnlund et al., 2004).

Based on the few previous studies conducted in other species and the greater Cu exposure dairy-beef calves face, it is possible that excess Cu status hinders vaccine response, but this has not yet been examined in dairy-beef calves. Thus, the objective of this study was to determine the impact of liver Cu concentration on antibody production in response to a modified live respiratory vaccine (Bovilis Vista 5, Merck Animal Health, Madison, NJ). We hypothesized that steers with excess liver Cu concentrations will have decreased antibody titer production compared with those with adequate liver Cu concentrations.

Sixty-four weaned dairy-beef crossbred steers (95 ± 7.0 kg, approximately 8 wk of age) were purchased from a single grower, but multiple source dairies, and delivered to the Iowa State University Beef Nutrition Farm (Ames, IA) on May 18, 2023. Upon arrival, steers were dewormed (Safe-Guard oral suspension, Merck Animal Health) and blocked by BW into 8 pens (8 steers per pen). Using a computer-generated random number sequence, one pen from each weight block was randomly assigned to one of 2 target liver Cu statuses: adequate Cu (**ADE**) or high Cu (**HCU**). To achieve target Cu status, all steers were fed a pelleted diet containing 20 or 10 mg Cu/kg diet DM for ADE and HCU, respectively, plus ad libitum hay. This pelleted diet was fed for 47 d before steers were gradually transitioned to a TMR (Table 1), which was supplemented with 0 or 10 mg Cu from Cu sulfate/kg diet DM for ADE and HCU, respectively. Due to health concerns, all steers were treated with Draxxin (Zoetis, Parsippany, NJ) 25 d after arrival. Steers were administered a zeranol implant (Ralgro, Merck Animal Health) 39 d after arrival. Liver biopsies were collected from all animals 81 d postarrival to confirm Cu status aligned with treatment assignments; n = 13 and n = 15 under ADE and HCU, respectively, were chosen for enrollment in the study. Because liver Cu data from individual animals were needed to appropriately enroll animals to the study, researchers were not blinded to treatment.

**Table 1:** Diet composition of common TMR fed to both treatments starting 47 d after arrival

Item	Value
Ingredient, % of diet DM	
Hay	15
Corn	15
Corn silage	15
Dried distillers grains	18.06
Sweet Bran^1^	35
Cu premix^2^	5
Trace mineral premix^3^	0.0204
Limestone	1.5
Salt	0.31
Vitamin A and E premix^4^	0.1
Rumensin 90^5^	0.0135
Analyzed composition	
CP,^6^ %	18.4
NDF,^6^ %	33.1
Ether extract,^6^ %	5.2
S,^7^ %	0.31
Mo,^7^ mg/kg DM	0.97
Cu,^8^ mg/kg DM	4.8
Zn,^8^ mg/kg DM	67.5
Fe,^8^ mg/kg DM	211

1 Branded wet corn gluten feed (Cargill Milling, Blair, NE).

2 Copper treatments were included as a dried distillers grains–based premix that replaced dried distillers grains in the diet. Treatments included no supplemental Cu (ADE) and 10 mg Cu/kg diet DM (HCU).

3 Trace mineral premix was formulated to supplement all trace minerals other than Cu at NASEM (2016) recommendations.

4 Vitamin A and E premix provided 2,200 IU of vitamin A and 25 IU of vitamin E/kg diet DM.

5 Granulated Monensin (Elanco Animal Health, Greenfield, IN), formulated to provide 27 mg of Monensin/kg of diet DM.

6 Analysis of ADE TMR composite by Dairyland Laboratories (Arcadia, WI).

7 Analysis of ADE TMR composite by the Iowa State University Veterinary Diagnostic Laboratory (Ames, IA).

8 Analyzed Fe, Zn, and Cu represent ADE dietary treatment with no supplemental Cu and were analyzed via inductively coupled plasma-optical emission spectrometry (ICP Optima 7000 DV, Perkin Elmer, Waltham, MA). Copper treatments were supplemented in addition to Cu in the basal diet.

Day 0 of the vaccine challenge began 89 d after steers initially arrived, on August 15, 2023. Liver Cu for ADE averaged 291 ± 24 mg/kg DM (range 240–376 mg/kg DM), and HCU averaged 665 ± 23 mg Cu/kg liver DM (range 519–893 mg/kg DM). Steers were weighed and administered a respiratory vaccine (Bovilis Vista 5, Merck Animal Health) as well as 2 mL of an ovalbumin vaccine subcutaneously (EndoFit OVA, InvivoGen, San Diego, CA) containing 2 mg of ovalbumin glycoprotein adjuvanted with a 15% vol/vol solution of Montanide Gel PR01 (Seppic, Courbevoie, France). Steers were weighed and boostered with both vaccinations 21 d later.

Weekly TMR samples were collected and dried in a forced-air oven at 70°C for 48 h to determine DM content, after which they were ground through a 2 mm screen (Retsch Zm100 grinder; Retsch GmBH, Haan, Germany). Samples were composited by month for further analysis. Liver biopsies were collected on d −7 as previously described by Engle and Spears (2000) and were transported on ice to the laboratory, after which they were stored at −20°C until further analysis. Blood was collected on d 0, 7, and 49 via jugular venipuncture into one serum separator tube and one K_2_EDTA plasma tube (Becton Dickenson, Rutherford, NJ). Blood was transported to the laboratory and centrifuged at 1,000 × *g* for 20 min at 4°C, and serum and plasma were harvested and stored at −80°C and −20°C, respectively, until further analysis.

Copper concentrations in plasma, liver, and monthly TMR composites were determined via inductively coupled plasma-optical emissions spectrometry (**ICP-OES**; ICP Optima 7000 DV, Perkin Elmer, Waltham, MA). Plasma samples were prepared following methods outlined in Pogge and Hansen (2013). Briefly, plasma was diluted 1:7 in 5% trace mineral grade nitric acid before centrifugation at 760 × *g* for 10 min at 4°C. The resulting supernatant was collected for Cu analysis via ICP-OES. Liver and TMR samples were dried at 70°C in a forced air oven before digestion. Approximately 0.25 g of dried liver or 1.0 g of dried TMR composite was digested with 5 mL or 10 mL of trace mineral grade nitric acid, respectively, using a closed-vessel microwave digestion system (MARS Xpress, CEM Corporation, Matthews, NC). Following digestion, samples were brought to a final volume of 25 mL (liver) or 50 mL (TMR) with deionized water before Cu analysis via ICP-OES.

Serum was analyzed by the Iowa State University Veterinary Diagnostic Laboratory for bovine respiratory syncytial virus (**BRSV**), bovine herpesvirus-1 (**BHV1**), bovine viral diarrhea virus-1 (**BVDV1**), and bovine viral diarrhea virus-2 (**BVDV2**) neutralizing titers. Serum was also analyzed for haptoglobin via commercial ELISA (Immunology Consultants Laboratory Inc., Portland, OR), where haptoglobin was quantified through antigen-antibody binding detected via colorimetric reaction, and haptoglobin concentrations of unknown samples calculated using a standard curve of known concentrations provided by the manufacturer.

Ovalbumin (**OVA**) served as a model antigen to evaluate the immune response independent of factors such as maternal antibodies. Response to ovalbumin vaccination was analyzed via indirect ELISA using methods adapted from Rivera et al. (2002) and Chang et al. (1996), where antigen-antibody binding was detected through colorimetric reaction. In brief, 96-well plates (Immulon 4HBX Nunc; Thermo Fisher, Waltham, MA) were coated with 100 µL of OVA antigen per well, diluted to 2 µg/mL in bicarbonate buffer, overnight at 4°C. The following day, plates were blocked with Starting Block (Thermo Fisher) at 150 µL per well for 1 h, after which plates were washed with 5% Tween 20 in PBS. Serum samples were diluted 1:50 in 3% BSA-PBS solution (Sigma-Aldrich, St. Louis, MO) and added to the wells in duplicate, along with blanks and a positive and negative pool at 100 µL per well. After a 2-h incubation, plates were washed and 100 µL of rabbit anti-bovine IgG-HRP (Bethyl Laboratories, Montgomery, TX), diluted 1:10,000, were added to each well and incubated for 30 min. Plates were then washed, and 100 µL of TMB substrate (One-Step Ultra TMB; Thermo Fisher) was added to each well. After 15 min of incubation, the reaction was stopped by adding 50 µL of H_2_SO_4_ to each well. Absorbances were read at 450 and 605 nm; 605 nm absorbances were subtracted from 450 nm absorbances to correct for background. Antibody production was determined by calculating the ratio between each sample's absorbance and the absorbance of a positive control.

Data were analyzed as a completely randomized design in the MIXED procedure of SAS 9.4 (SAS Institute Inc., Cary, NC). Because liver Cu concentration was determined from each animal, steer was the experimental unit. All titer, OVA response, and haptoglobin data were transformed using the natural logarithm to meet assumptions for normality based on the Shapiro–Wilk test and analyzed with the fixed effect of Cu treatment and day as repeated. Interactions of Cu treatment, day, and treatment × day were analyzed. Cook's D statistic was used to test for outliers with a cutoff value of 0.5. Six single-time point samples were removed from haptoglobin analysis due to being high outliers (3 HCU, 3 ADE). One steer (ADE) was removed from the trial due to unrelated health complications and was excluded from all data analysis. Data shown are back-transformed LSM and SEM.

Before vaccination, liver Cu averaged 291 ± 24 and 665 ± 23 mg Cu/kg liver DM for ADE and HCU, respectively. Plasma Cu concentrations are shown in Figure 1A and were affected by day (*P* < 0.01), where plasma Cu did not differ between d 0 and 21 (*P* = 0.17) and was lesser on d 49 (*P* < 0.01). Plasma Cu concentration was not affected by treatment or treatment × day (*P* ≥ 0.39).

**Figure 1:**
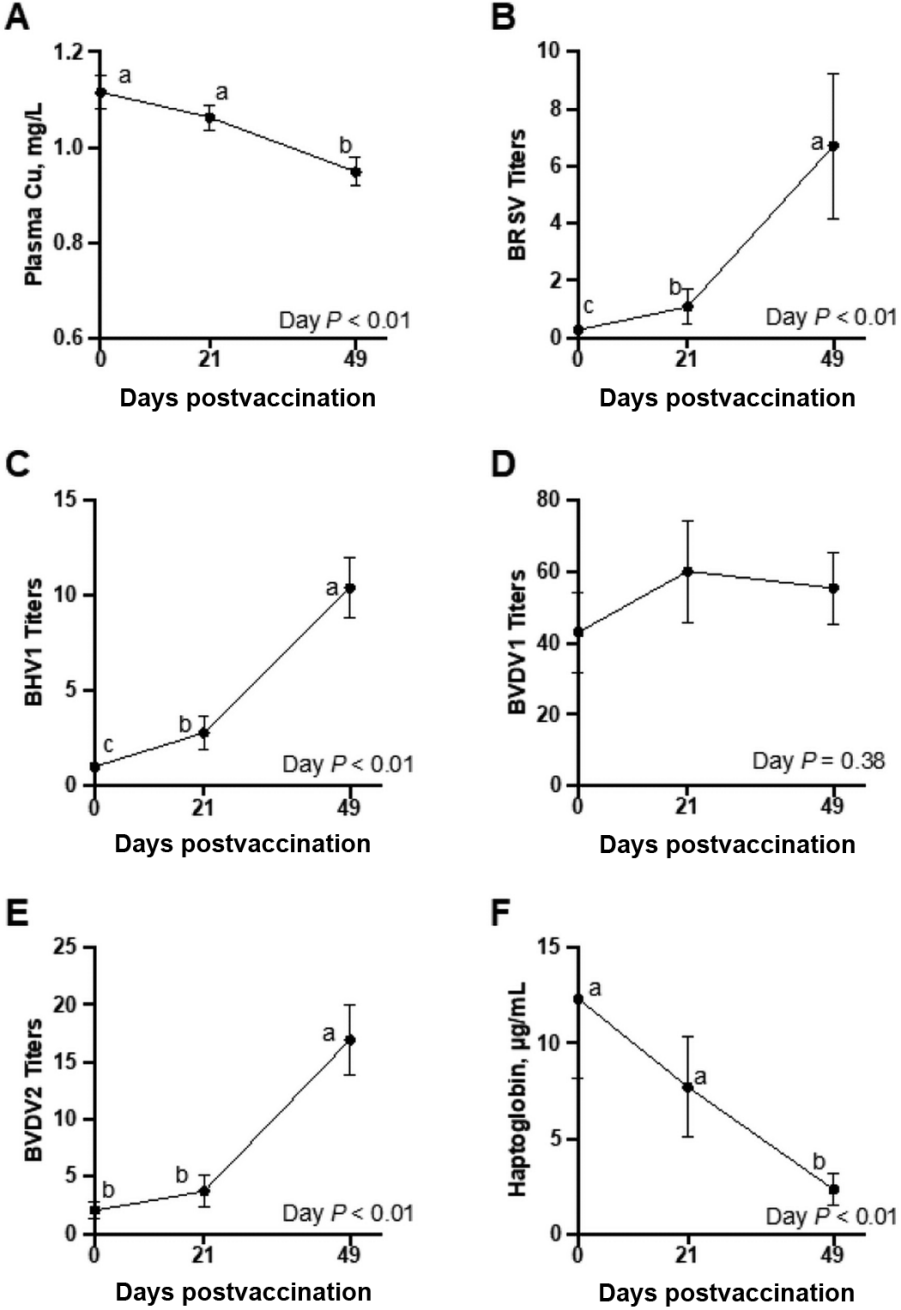
The effect of day relative to vaccination (Bovilis Vista 5 SQ, Merck Animal Health, Madison, NJ) on (A) plasma Cu concentration, (B–E) serum neutralizing antibody titers (displayed as reciprocal dilution factors), and (F) serum haptoglobin concentration in dairy × beef crossbred steers. Within a panel, data points with unlike lowercase letters differ by day (*P* ≤ 0.05). Error bars represent SEM. No day effect was noted for BVDV1 neutralizing titers, and no significant treatment or treatment × day effects were noted for any viral titers, haptoglobin, or plasma Cu.

Serum neutralizing antibody titers for BRSV, BHV1, BVDV1, and BVDV2 are shown in Figure 1B–E, respectively. All titer data are expressed as the reciprocal of the highest serum dilution yielding a positive result in the assay. None of the titers measured were affected by treatment or treatment × day (*P* ≥ 0.12). Bovine respiratory syncytial virus neutralizing titers averaged 0.27 ± 0.11 on d 0, 1.08 ± 0.60 on d 21, and 6.72 ± 2.54 on d 49 (day *P* < 0.01). Titers for BHV1 also increased with sampling day (*P* < 0.01), where titers were 1.02 ± 0.022 on d 0, 2.80 ± 0.89 on d 21, and 10.38 ± 1.58 on d 49. Bovine viral diarrhea virus 2 was affected by day (*P* < 0.01) where titers were similar on d 0 and 21 (2.04 ± 0.77 and 3.71 ± 1.40; *P* = 0.14) and had increased by d 49 (16.95 ± 3.12; *P* < 0.01). Titers for BVDV1 were not affected by treatment, day, or treatment × day (*P* ≥ 0.38), and were 42.96 ± 11.17 on d 0, 60.06 ± 14.23 on d 21, and 55.40 ± 10.12 on d 49. Given the presence of titers on d 0 and the lack of response, it appears these steers were previously exposed to BVDV1.

The relative-to-positive ratios for ovalbumin vaccine response are presented in Figure 2. There was a treatment × day effect (*P* = 0.04) where both treatments were similar on d 0 (*P* = 0.16), and on d 21, HCU exhibited a transiently greater response than ADE (*P* < 0.01). By d 49, both treatments were similar (*P* = 0.52) and were greater than d 0 and 21 (*P* ≤ 0.04).

**Figure 2:**
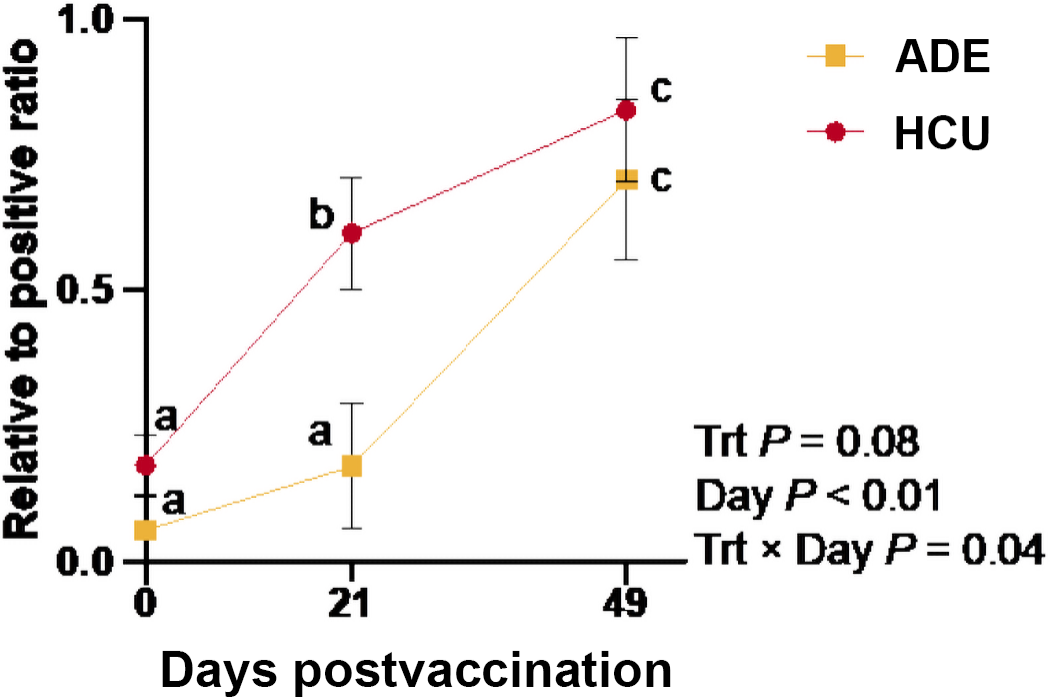
Effect of liver Cu × day (*P* = 0.04) relative to ovalbumin vaccination on ovalbumin antibody production in lightweight dairy × beef crossbred steers. Results were obtained by calculating the ratio of the absorbance of each sample to a positive control. Data points with different lowercase letters differ by treatment × day (*P* ≤ 0.05). Error bars represent SEM.

Serum haptoglobin concentrations are presented in Figure 1F and were not affected by treatment or treatment × day (*P* ≥ 0.47) but were affected by day (*P* < 0.01). Haptoglobin concentrations were similar on d 0 and 21 (*P* = 0.26) and were decreased on d 49 (*P* < 0.01). Notably, a great deal of variation was present within this dataset, especially on d 21, and this may have affected the ability to detect treatment effects.

Dairy-beef cattle are frequently exposed to greater amounts of Cu than native beef cattle due to management practices in the dairy industry. Research across various species indicates excess Cu contributes to liver damage and inflammation (Humann-Ziehank et al., 2001; Smedley et al., 2009), potentially altering immune function. The upper threshold for “adequate” liver Cu concentrations in cattle is 600 mg Cu/kg liver DM (Kincaid, 1999). In the present study, liver Cu concentrations in the HCU treatment ranged from 519 to 894 mg Cu/kg liver DM.

Excess Cu did not result in increased haptoglobin, an inflammatory marker, in the HCU treatment. However, haptoglobin concentrations decreased steadily over time in both treatments, which could indicate a pro-inflammatory event before d 0 that was resolving during the vaccination period. The liver biopsy conducted on d −8 may have contributed to this inflammation, though the specific cause is unknown. Haptoglobin is only one marker of inflammation, and the lack of difference between treatments does not preclude effects of Cu status on other inflammatory pathways that were not examined in the present study. Further examination of inflammation and oxidative stress markers is necessary to better characterize the relationship between excess Cu and inflammation.

Previous work in humans has shown that excess Cu reduces the immune response to influenza vaccination (Turnlund et al., 2004). In contrast, both treatments in the present study responded equally well to vaccination against BRSV, BHV1, and BVDV2. The high titers for BVDV1 before vaccination indicate that these cattle were likely previously exposed to BVDV1 and did not have increased antibody production in response to vaccination. Because these calves may have had previous viral exposure or may have still possessed maternal antibodies, an ovalbumin vaccine was administered to evaluate antibody production to a foreign antigen. Interestingly, calves in the HCU treatment appeared to have an earlier antibody response to ovalbumin than those in the ADE group, as evidenced by the greater relative-to-positive ratio on d 21. However, this difference was not sustained, as ratios were similar between treatments by d 49.

Although no differences in haptoglobin concentrations were observed between ADE and HCU, it remains plausible that calves in the HCU treatment experienced liver inflammation. Such inflammation could have triggered the release of pro-inflammatory cytokines, including IL-6, which is known to enhance B-cell differentiation and recruitment, thereby promoting increased antibody production (Dienz et al., 2009). Accelerated B-cell differentiation and recruitment caused by inflammation could explain the transiently greater antibody titers observed in HCU steers on d 21. Alternatively, Cu status might influence immune kinetics through antigen presentation. Copper ions have been shown to enhance the expression of antigen-presenting markers on murine dendritic cells in vitro (Schuhladen et al., 2020), potentially altering T-cell priming and thus contributing to the earlier ovalbumin antibody production observed in the high treatment of the present study. Further, patients with Wilson's disease, a genetic disorder characterized by hepatic copper accumulation, exhibit greater concentrations of IgG, IgM, and antibodies against Kunin's common antigen (Członkowska and Milewski, 1976). Leukocytes of Wilson's disease patients have impaired bactericidal ability, and free Cu ions may inhibit cell-mediated immunity. The authors hypothesize Cu may have an inhibitory effect on T-cells, disturbing their regulation of B-cells, which may lead to overactivation of B-cells. This may be an intrinsic reaction by the B-cells to maintain some degree of immune function in the absence of T-cells. It is possible that a similar effect was occurring in the present study where excess Cu resulted in hyperactivated B-cells in the HCU steers, causing a shift in the dynamics of antibody production due to altered regulation. This shift might be antigen dependent given this interaction was not observed with viral antibodies.

In conclusion, contrary to our hypothesis, excess liver Cu did not affect the response of dairy-beef steers to a respiratory vaccine; however, steers with excess liver Cu appeared to exhibit an accelerated initial response to the model antigen, ovalbumin, although this effect was transient and did not result in greater overall antibody production by d 49. This timing difference may suggest excess Cu alters immune kinetics, and more research is needed to better understand the role of excess liver Cu in modulating the immune response and its impact on disease outcomes.
